# Antioxidant and Anti-Inflammatory Properties of Anthocyanins Extracted from *Oryza sativa* L. in Primary Dermal Fibroblasts

**DOI:** 10.1155/2019/2089817

**Published:** 2019-07-31

**Authors:** Pakhawadee Palungwachira, Salunya Tancharoen, Chareerut Phruksaniyom, Sirinapha Klungsaeng, Ratchaporn Srichan, Kiyoshi Kikuchi, Thamthiwat Nararatwanchai

**Affiliations:** ^1^School of Anti-Aging and Regenerative Medicine, Mae Fah Luang University, Chiang Rai, Thailand; ^2^Department of Pharmacology, Faculty of Dentistry, Mahidol University, Bangkok, Thailand; ^3^Department of Pharmacology, Faculty of Medicine, Khon Kaen University, Khon Kaen, Thailand; ^4^Oral Tissues, Cells and Molecular Biology Analysis and Research Center, Faculty of Dentistry, Mahidol University, Bangkok, Thailand; ^5^Division of Brain Science, Department of Physiology, Kurume University School of Medicine, Fukuoka 830-0011, Japan

## Abstract

Flavonoids are naturally active substances that form a large class of phenolic compounds abundant in certain foods. Black rice (*Oryza sativa* L.) contains high levels of anthocyanin polyphenols, which have beneficial effects on health owing to their antioxidant properties. The breakdown of collagenous networks with aging or skin deterioration results in the impairment of wound healing in the skin. Accordingly, reviving stagnant collagen synthesis can help maintain dermal homeostasis during wound healing. This study presents an assessment of the cellular activity of anthocyanins (ANT) extracted from *Oryza sativa* L., providing information necessary for the development of new products that support natural healing processes. The relative composition of ANT from *Oryza sativa* L. was determined by high-performance liquid chromatography/diode array detection. ANT promoted the migration of rat dermal fibroblasts (RDFs) and demonstrated antioxidant properties. ANT increased the mRNA expression of *collagen type I alpha 2* (*COL1A2*) and upregulated type I collagen protein levels in H_2_O_2_-stimulated RDFs without cytotoxicity. Compared with the untreated group, treatment of RDFs with ANT in the presence of H_2_O_2_ led to the activation of signaling pathways, including the extracellular signal-regulated protein kinases 1 and 2 (ERK1/2) and Akt, whereas it significantly (*p* < 0.001) inhibited the phosphorylation of I*κ*B*α* and suppressed the activation of the nuclear factor-kappa B (NF-*κ*B) subunits, p50 and p65, which are transcription factors responsible for inflammation. Taken together, our findings suggest that ANT from *Oryza sativa* L. have anti-inflammatory properties and antiaging potential by modulating type I collagen gene expression and suppressing H_2_O_2_-induced NF-*κ*B activation in skin fibroblasts.

## 1. Introduction

Black rice (*Oryza sativa* L.) has been widely consumed since ancient times in parts of Asia including present-day Thailand. In an attempt to know its effective beneficial health components, many investigators have aimed on the characteristics of flavonoids, a large class of phenolic compounds that is found plenty in foods including black rice. The most prominent flavonoids in *O. sativa* L. are the anthocyanins (ANT), which are ubiquitous coloring agents in plants accountable for the purple, red, and blue hues of many types of fruit, vegetables, cereal grains, and flowers [1]. *O. sativa* L. has been considered as a health-supporting food given its abundance of ANT [2]. Many studies have revealed that ANT have positive effects on a variety of health conditions [3]. One reason for this is their anti-inflammatory properties, which occur via effects on collagen synthesis [4, 5].

Proanthocyanidins are a class of biologically active polyphenolic bioflavonoids known to be synthesized by many plants. ANT-demonstrated benefits include protection against diabetes [6, 7], impressive reduction of blood pressure, enhancement of vision [8, 9], and strong anti-inflammatory effects [10, 11]. These phenolic compounds also facilitate dermal and oral wound healing [11, 12]. Fibroblast proliferation influences the formation of granulation tissue, collagen synthesis, and wound contraction. The granulation tissue generated by fibroblasts immersed in a loose matrix of collagen fibers consisting primarily of type I collagen is accumulated in the wound base [13]. In addition, ANT stimulate wound healing while suppressing superfluous inflammation by inducing vascular endothelial growth factor (VEGF) production in fibroblasts and keratinocytes [11].

Oxidative stress has been implicated to damage various cellular portions involving lipids, proteins, and nucleic acids through oxidation by reactive oxygen species (ROS) such as hydrogen peroxide (H_2_O_2_), hydroxyl radical (OH^−^), and superoxide anion radical (O_2_^−^) [14]. ROS are very harmful to the wound healing process owing to their harmful effects on extracellular matrix components, including collagens, and on keratinocyte functions and the damage they induce in dermal fibroblasts [15]. Oxidative process involved in the pathogenesis of many diseases suggests that antioxidants may play an important role for the treatment in these conditions. Topical treatment with compounds that have antioxidant properties has been shown to significantly enhance the wound healing process and protect tissues against oxidative damage [16]. The free-radical-scavenging and antioxidant capacities of ANT pigments are introduced as the most highly recognized modes of action for interposing with numerous human therapeutic targets [17]. Moreover, with their anabolic effects *in vivo*, there is substantial verification suggesting that these phytochemicals have positive effects on wound healing [18]. In a previous study, ANT were found to promote wound healing by preventing oxidative damage [19]; however, to the best of our knowledge, the mechanisms by which ANT extracted from *O. sativa* L. act in modulating collagen formation and inflammation in skin fibroblasts have not been investigated.

The transcription factor, nuclear factor-*κ*B (NF-*κ*B), regulates a large number of genes that are involved in inflammation and immune responses. This stress-regulated transcription factor has multiple effects on the immune system [20] and can modulate the expression of proinflammatory genes encoding nitric oxide synthase, cytokines, and chemokines [21]. NF-*κ*B overstimulation has been shown to result in cellular and DNA damage by ROS production [22, 23]. In addition, one of the key elements in the inflammatory process is the activation and translocation of NF-*κ*B from the cytosol to the nucleus. Subunits p50 and p65 which are the classical NF-*κ*B heterodimer exist as the potent activator of gene expression [24]. The activation of variable extracellular stimuli such as environmental stress including UV light and stimuli such as interleukin- (IL-) 1, tumor necrosis factor- (TNF-) *α*, viruses and endotoxins, and I*κ*B will be phosphorylated and degraded by the *26S* proteasome. This gives rise to the translocation of p50/p65 heterodimer to the nucleus, where it links to its responsive elements present in a variety of genes [25]. In consequence, the various combinations of NF-*κ*B subunits may organize the complicated regulatory pathway of NF-*κ*B like orchestral performance. We hypothesized that ANT extracted from black rice may have anti-inflammatory and antioxidant effects associated with collagen production in skin fibroblasts. In this study, we showed that ANT extracted from *O. sativa* L. have strong antioxidant and anti-inflammatory activities in rat primary dermal fibroblasts. ANT protect against the effects of H_2_O_2_-induced collagen degradation, by suppressing I*κ*B phosphorylation and the activation of NF-*κ*B p50/p65.

## 2. Materials and Methods

### 2.1. Chemicals, Reagents, and Antibodies

ERK1/2 (pT202/Y204+Total) ELISA Kit, Akt (pS473+Total) ELISA kit, Rat IL-6 ELISA Kit, Abs for isotype control IgG, rabbit polyclonal Abs for NF-*κ*B p50, p65, phosphor-Y42 I*κ*B*α*, and Histone H3, mouse monoclonal Abs for *β*-actin, anti-rabbit IgG (HRP), and anti-mouse IgG (HRP), and anti-rabbit fluorescein isothiocyanate (FITC) secondary Abs were obtained from Abcam (Cambridge, MA, USA). MTT (3-(4,5-dimethylthiazol-2-yl)-2,5-diphenyl tetrazolium bromide) solution was obtained from Promega (Madison, WI, USA). A Total Collagen Assay (Colorimetric) Kit was purchased from BioVision (Milpitas, CA, USA). Phorbol 12-myristate 13-acetate (PMA) was purchased from Sigma-Aldrich Inc. (St. Louis, MO, USA). A Cell Fractionation Kit was purchased from Abcam. Unless stated otherwise, all other reagents were purchased from Sigma-Aldrich Inc.

### 2.2. Plant Material and Extraction

Black rice was obtained from Chachoengsao Province, Thailand, and subjected to ANT extraction as previously reported [26]. ANT were extracted in ethanol (60/40, *v*/*v*%), concentrated using a Büchi B-490 rotary evaporator (Büchi Labortechnik AG, Flawil, Switzerland) and lyophilized with a freeze dryer (Labconco Corp., Kansas City, MO, USA). The crude extract was stored at room temperature (RT).

### 2.3. Determination of Flavonoids and ANT Composition by High-Performance Liquid Chromatography (HPLC)

The composition of ANT extract was identified using HPLC/diode array detection (DAD) as previously reported [27]. A Waters LC-MSD 1525 series equipped with a UV detector and Agilent Zorbax SB-C18 column was used. The solvents were aqueous 2% formic acid and acetonitrile : water (1 : 1 *v*/*v*) containing 2% formic acid. Injection volumes were 15 *μ*L. The separated ANT components were measured at 516 nm and were identified based on their retention times. ANT were quantified by UV-visible (Vis) spectroscopy as previously described [28]. The model reaction solution was diluted with 0.01% HCl in distilled water. Absorbance was measured using a Genesys 10 UV spectrophotometer (Thermo Fisher Scientific, Grand Island, NY, USA).

### 2.4. Estimation of Total Phenolic Content

The total phenolic content was determined using Folin-Ciocalteu reagent (FRC) as previously described [29], with minor modifications. The absorbance of the mixture was measured at 765 nm using a UV-Vis Genesys 10 UV spectrophotometer. A standard curve was plotted using gallic acid (0.07–10 mg/mL in methanol; Sigma-Aldrich) as a standard. The total phenolic content was expressed as gallic acid equivalents (mM GAE/gFW).

### 2.5. Cell Culture and Treatment

Rat primary dermal fibroblasts (RDFs), growth media, and passaging solutions were purchased from Cell Applications (San Diego, CA, USA). Cells were maintained in Culture Complete Growth Medium and penicillin/streptomycin solution in a humidified incubator with 5% CO_2_ at 37°C. To eliminate any possible side effects of growth factors, cells were cultured in serum-free medium for 24 h before treatment with ANT extract.

### 2.6. Cell Viability Test

Cell viability was measured using the MTT assay in accordance with a previously described method [30]. Briefly, after incubation of the cells (1 × 10^5^ cells/mL) in 96-well plates with various concentrations of ANT (5, 10, 25, and 50 *μ*g/mL) for 24 h, they were incubated with MTT (0.5 mg/mL). Three hours later, dimethyl sulfoxide (DMSO) was added and the absorbance of the solution was measured at 570 nm with an automatic microplate reader (ImmunoMini NJ-2300; InterMed, Tokyo, Japan). The cell survival percentage was calculated using DMSO-treated cells as a standard. The experiments were repeated three times with five samples for each group.

### 2.7. Determination of Total Antioxidant Capacity and Copper Ion Reduction Activity

The Total Antioxidant Capacity (TAC) Assay Kit (Cell Biolabs OxiSelect™, San Diego, CA, USA) was used to measure the antioxidant capacity of ANT from black rice. The TAC Assay is based on the reduction of copper (II) to copper (I) by antioxidants such as uric acid. Briefly, RDFs were treated with 5–50 *μ*g/mL ANT and stimulated with 0.3 mM H_2_O_2_ for 24 h. The supernatant was dispensed in a 96-well microtiter plate, after which 180 *μ*L of 1× reaction buffer was added to each well and mixed. An initial reading at 490 nm was taken for each sample. Then, 50 *μ*L of 1× copper ion reagent was added and incubated for 5 min on an orbital shaker. Next, 50 *μ*L of stop solution was added to terminate the reaction, and the plate was subjected to additional measurement of absorption at 490 nm using a spectrophotometer (Epoch, BioTek, Winooski, VT, USA) with the Gen 5 data analysis software interface. All determinations were performed in triplicate, and results were averaged. The copper ion reduction activity of each sample with H_2_O_2_ was then calculated as the percent increase in ion reduction.

### 2.8. Monitoring Cell Migration by Real-Time Cell Analysis (RTCA)

The rate of cell migration was monitored in real time with the xCELLigence system using CIM plates (ACEA Biosciences Inc., San Diego, CA, USA). Video clips of several experimental tests were observed to determine the optimal seeding density of the RDFs (Supplementary Movie 1). The optimal cell density at each time point for these migration profiles was 5000 cells/well, and hence, this number of RDFs was seeded in each well. Cells were serum-starved for approximately 24 h. The upper chamber (UC) of the CIM plates was coated with 1 *μ*g/*μ*L fibronectin. A total of 5000 cells were seeded in the UC of each well in a serum-free medium. Fresh medium was added to the lower chamber (LC) of each well, and cells were stimulated with 5–25 *μ*g/mL ANT. The CIM plates were left in an incubator for 1 h to allow cells to attach. The impedance value of each well was automatically monitored by the xCELLigence system at 6, 12, and 24 h and expressed as CI (cell index). Data of cell migration were normalized at 30 min. The CI was calculated automatically by the RTCA Software Package 2.1.0. The CI calculation is based on the following formula:

(1) $$\begin{array}{c} \text{CI}=\frac{\left(Z_{i}-Z_{0}\right)}{15\varsigma }, \end{array}$$

CI calculation used to measure the relative change in electrical impedance, representing the cell status (equation (1)). The unit of the impedance is ohm (*Ω*):

- *Z*_*i*_ = impedance at an individual point of time during the experiment
- *Z*_0_ = impedance at the start of the experiment

Normalized CI was calculated as CI at a given time point divided by the CI at the normalization time point. The rate of cell migration was determined by calculating the slope of the line between two given time points.

### 2.9. Cell Migration Assay Using a Boyden Chamber

To analyze the migration of RDFs, a modified Boyden chamber with 24-well Nunc™ cell culture inserts containing a polycarbonate filter with 8 *μ*m pores was used. The inner layer of Transwell inserts was coated with 1 mg/mL Matrigel (BD Biosciences, Palo Alto, CA, USA) overnight at 4°C. Fibroblasts were placed in the inner well of the Transwell at 5 × 10^4^ cells/well in 200 *μ*L of Dulbecco's modified Eagle's media containing 1% FBS. Various concentrations of ANT (5, 10, and 25 *μ*g/mL) in 750 *μ*L of culture medium were added to the bottom chamber. After incubation at 37°C for 24 h, the inner well was wiped with a cotton swab to remove nonmigrating cells. The cells were fixed using 100% (*w*/*v*) methanol at RT for 10 min, after which the membrane was transferred onto a cover slide. Cells were stained with 10% Giemsa stain and assessed by light microscopy. Fifteen high-power fields (hpf) per membrane were counted, and migration was expressed as the mean number of cells/hpf ± SEM. To ensure reproducibility, each condition was tested in three separate wells. For each experiment, cell viability was assessed by trypan blue exclusion before using cells in the assay. In all cases, cell viability exceeded 90%.

### 2.10. RNA Extraction, cDNA Synthesis, and Quantitative Reverse Transcription-PCR (RT-qPCR)

RDFs were treated with 5, 10, and 25 *μ*g/mL ANT or PMA with or without 0.3 mM H_2_O_2_ for 4 and 10 h. After removing the supernatant and collecting the cell pellets, total RNA was isolated with FavorPrep Tissue Total RNA Mini Kit (Favorgen Biotech, Pingtung, Taiwan) in accordance with the manufacturer's instructions. The absorbance of nucleic acids was measured using a Genesys 10 UV spectrophotometer (Thermo Fisher Scientific). One microgram of RNA was subjected to DNase I digestion, followed by reverse transcription using a DNase I, RNase-free kit (Thermo Fisher Scientific). Total RNA (1 *μ*g) was reverse-transcribed into cDNA using iScript™ Select cDNA Synthesis Kit (Bio-Rad, Hercules, CA, USA) in a total volume of 20 *μ*L using oligo-dT primers. cDNA (<20 ng/well) was used as a template in qPCR reactions with oligonucleotides specific for the genes of interest (Table 1). PCR amplification was conducted in a total volume of 20 *μ*L including 20 ng of the template cDNA, 7.2 *μ*L of PCR-grade water (Welgene, Daegu, South Korea), 10 *μ*M of each primer, and 10 *μ*L of 2× KAPA SYBR® FAST qPCR Master Mix Universal from KAPA SYBR® FAST qPCR Kit Master Mix (2×) Universal (KAPA Biosystems, Selangor, Malaysia). A nontemplate control and an RNA sample without reverse transcription for each sample were used as control for potential DNA contamination. Unless stated otherwise, the temperature protocol for PCR amplification was as follows: enzyme deactivation at 95°C for 3 min, followed by 40 cycles of denaturation at 95°C for 3 s plus combined annealing/extension at 60°C for 30 s. Melting curve analysis was then performed for 10 s at 95°C with 0.5°C increments between 65°C and 95°C. All qPCR reactions were performed in triplicate. Relative quantification was determined using the CFX Manager Software (version 2.0; Bio-Rad) measuring SYBR green fluorescence.

### 2.11. Total Collagen Estimation by Colorimetric Assay

RDFs cultured in 96-well plates at a density of 3 × 10^4^ cells/well in 200 *μ*L were pretreated with 5, 10, and 25 *μ*g/mL ANT for 2 h with or without 0.3 mM H_2_O_2_, followed by incubation for 24 h. Estimation of the collagen level was carried out using a Total Collagen Assay Kit, in accordance with the manufacturer's instructions. Each sample was homogenized in 100 *μ*L of ddH_2_O. Briefly, each cell lysate was hydrolyzed with concentrated HCl at 120°C for 3 h, followed by vortexing and centrifugation at 10,000 × *g* for 3 min to remove the precipitate. A total of 10–30 *μ*L of each hydrolyzed sample was transferred to a 96-well plate and dried by evaporation at 70°C. Next, 100 *μ*L of Chloramine T reagent was added to each sample and incubated at RT for 5 min, after which 4-(dimethylamino)benzaldehyde (DMAB) reagent was added and incubated for 90 min at 60°C. Finally, hydroxyproline was oxidized to form a reaction intermediate and absorbance at 560 nm was determined in a microtiter plate using a microplate reader with an Epoch Microplate Spectrophotometer (Epoch; BioTek). The concentration of collagen was calculated from the collagen I standard curve provided with the kit.

### 2.12. Western Blot Analysis

The cells were plated in 10 cm dishes and pretreated with 10 or 25 *μ*g/mL ANT for 2 h before adding 0.3 mM H_2_O_2_, followed by incubation for 10 h. The cytoplasmic and nuclear extracts for immunoblotting were prepared using a Cell Fractionation Kit. Protein concentrations were measured and normalized with the BCA protein assay kit (Thermo Fisher Scientific). Each sample was subjected to electrophoresis on 12% SDS-polyacrylamide gels. Then, the protein was blotted onto nitrocellulose membranes. Membranes were incubated with primary antibodies against NF-*κ*B p50 (1 : 1000), NF-*κ*B p65 (1 : 1000), p-I*κ*B*α* Y42 (1 : 1000), *β*-actin (1 : 1000), and Histone H3 (1 : 500) overnight at 4°C. After washing, the membranes were incubated with secondary antibodies (HRP-conjugated goat anti-rabbit IgG or HRP-conjugated goat anti-mouse IgG) for 1 h. Immunoreactive proteins were detected with SuperSignal™ West Pico PLUS Chemiluminescent Substrate (Thermo Fisher Scientific). The intensity of the protein bands was quantified using ImageJ software.

### 2.13. Immunofluorescence Staining

RDFs were seeded on a four-well chamber slide (Nunc™ Lab-Tek™ II Chamber Slide™ System; Thermo Fisher Scientific) at a density of 2 × 10^4^ cells per well for 24 h. Cells were preincubated with ANT at a final concentration of 25 *μ*g/mL for 2 h, and NF-*κ*B activity was induced by 0.3 mM H_2_O_2_ for 24 h, after which cells were washed with PBS and then fixed with 100% methanol at −20°C for 20 min. Cells were blocked with 1% BSA in PBST (PBS+0.1% Tween 20) for 30 min at RT. After washing with PBS, the cells were incubated for 1 h at RT with anti-NF-*κ*B/p50 or anti-NF-*κ*B/p65 Ab (1 : 1000 dilution). Cells were also incubated with an isotype control rabbit monoclonal Ab as a negative control. After three washes with PBS, the cells were incubated for 1 h at RT with goat anti-rabbit IgG H&L conjugated with FITC (1 : 2500 dilution). Following three washes with PBS, the nuclei were counterstained with DAPI (Miltenyi Biotec, Bergisch Gladbach, Germany). The cells on the glass coverslips were embedded in a mounting medium (Aquamount; Lerner Laboratories, Pittsburgh, PA, USA) and then photographed with a fluorescence microscope (BX53 Digital Upright Microscope; Olympus, Tokyo, Japan).

### 2.14. Quantitative Estimation of Phosphorylated Akt and ERK1/2 and of IL-6 by Enzyme-Linked Immunosorbent Assay (ELISA)

RDFs were treated with 25 *μ*g/mL ANT or 0.3 mM H_2_O_2_ at various time intervals. After the treatment, the ANT and H_2_O_2_ effect on Akt and ERK1/2 phosphorylation was determined. Cells were then pretreated with 25 *μ*g/mL ANT for 2 h followed by a 30 min incubation with 0.3 mM H_2_O_2_ for Akt and 2 h for ERK1/2. The amounts of total and phosphorylated Akt and ERK1/2 in cell lysates were determined using Akt (pS473+Total) and ERK1/2 (pT202/Y204+Total) ELISA kits in accordance with the manufacturer's protocol. Briefly, cells were lysed in lysis buffer and then transferred to the ELISA plate coated with immobilized antibodies. The wells were washed, and antibodies from the kits were used to detect the phosphorylated and total forms of Akt and ERK1/2. For IL-6 determination, the protein release of IL-6 in RDF supernatants was evaluated with a commercial Rat IL-6 ELISA Kit in accordance with the manufacturer's instructions. The optical densities were measured at 450 nm with an Epoch Microplate Spectrophotometer (Epoch; BioTek).

### 2.15. Statistical Analysis

SPSS software (version 20.0; SPSS Inc., Chicago, IL, USA) was used for statistical analyses. All results are expressed as mean ± SD. The significance of differences between two groups was assessed using Student's *t*-test, and differences among multiple groups were assessed by one-way analysis of variance (ANOVA), followed by the Tukey-Kramer method. The level of significance was set at *p* < 0.05.

## 3. Results

### 3.1. Identification of Anthocyanins and Total Phenolic Content in *Oryza sativa* L.

**Figure 1:**
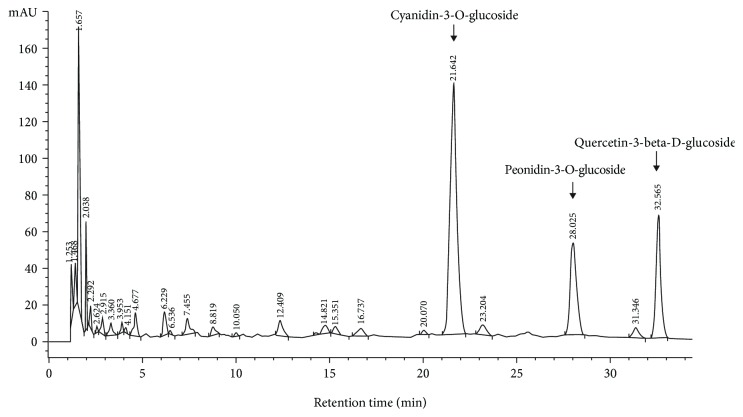
HPLC analysis of anthocyanins (ANT) in *Oryza sativa* L. Representative HPLC/DAD chromatograms of cyanidin-3-O-glucoside, peonidin-3-O-glucoside, and quercetin-3-beta-D-glucoside in the extracts at 516 nm. Peaks were detected with a retention time of 21.6, 28.0, and 32.5 min, respectively. Duplicate experiments were performed.

The ANT composition in *O. sativa* L. was determined by HPLC/DAD. The resulting chromatograms at 516 nm are shown (Figure 1). The chromatogram within the retention time of 21.6 min indicated the presence of cyanidin-3-O-glucoside (C3G), and the chromatogram within the retention time of 28.0 min was identified as peonidin-3-O-glucoside. Additionally, the retention time of 32.5 min indicated the presence of quercetin-3-beta-D-glucoside. The UV-Vis wavelength spectra quantification of total ANT showed that the phenolic-rich extract contained ~1704 mg/kg of total ANT (calculated as C3G equivalents), ~572.89 mg/kg of peonidin-3-O-glucoside, and ~728.37 mg/kg of quercetin-3-beta-D-glucoside. The total phenolic content was ~30 ± 1.5 mM gallic acid equivalent (gFW).

### 3.2. ANT Treatment Affects RDF Migration in a Dose-Dependent Manner

**Figure 2:**
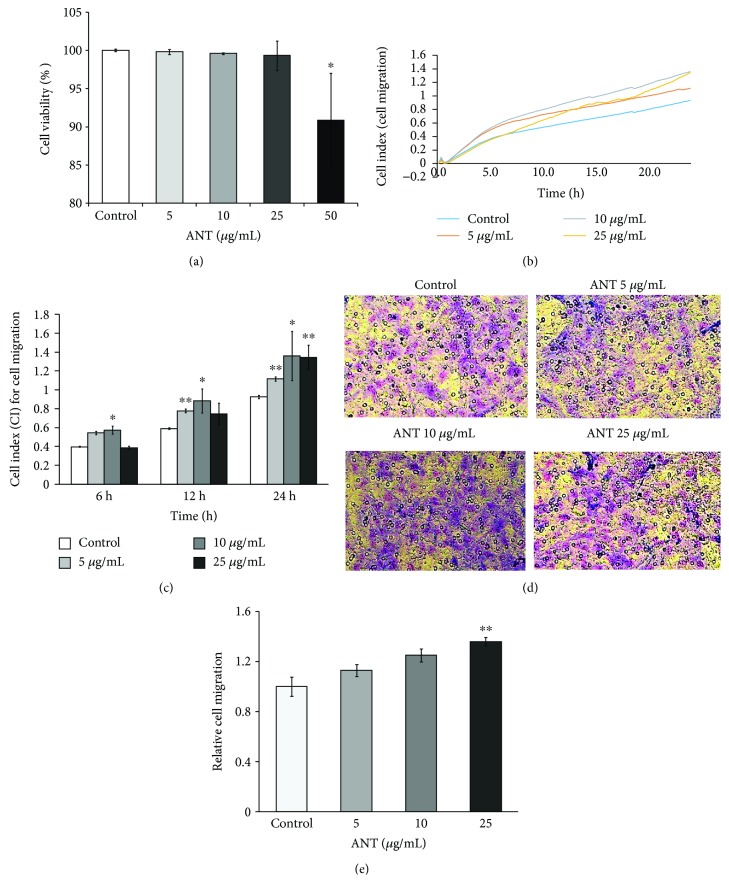
Effects of ANT on cell viability, migration, and chemotaxis of RDFs. RDFs were treated with or without ANT at 5–25 *μ*g/mL. (a) Cell viability was measured by the MTT assay. (b) Representative figure of CI values for cell migration over 24 h. (c) CI values for ANT at different time points following treatment with varying concentrations of ANT. (d) Boyden chamber assay demonstrating increased migration of RDFs incubated with ANT. Cells were allowed to migrate for 24 h, and then, they were fixed and stained with 10% Giemsa stain. The numbers of migrating cells were averaged from three ×10 field-of-view images. Original magnification ×100. (e) Results were quantified by counting the stained cells and are expressed relative to the mean number of cells randomly migrating in the control wells ± SEM. The results presented are from three independent experiments; ^∗^ *p* < 0.05 and ^∗∗^ *p* < 0.01 vs. control.

To examine cell viability in ANT-exposed RDFs, cells were treated for 24 h with various concentrations of ANT extracted from black rice. Cell viability was determined using the MTT assay. Our results demonstrated that 5, 10, and 25 *μ*g/mL ANT had no cytotoxic effects (Figure 2(a)). However, 50 *μ*g/mL ANT reduced cell viability to 90%. To minimize confounding effects due to reduced cell viability, ANT were used at the maximal noncytotoxic concentration, namely, 25 *μ*g/mL, in subsequent experiments. Based on the above results, we next investigated the effects of ANT on RDF migration, using the RTCA-DP xCELLigence system.

The migration profile of RDFs was indicated by CI values obtained within 24 h of testing. A marked increase in CI values was observed when cells were incubated with ANT at 5, 10, and 25 *μ*g/mL (Figure 2(b)). The results showed a significant increase in cell migration following stimulation with 5 *μ*g/mL (*p* < 0.05) and 10 *μ*g/mL (*p* < 0.01) ANT for 12 and 24 h (Figure 2(c)). However, a significant increase in cell migration (*p* < 0.01) was observed following stimulation with 25 *μ*g/mL ANT for 24 h. To confirm the effect of ANT on RDF chemotaxis, we determined the optimal concentration of ANT required to stimulate the migration of these cells using a Boyden chamber (Figure 2(d)). We found that 25 *μ*g/mL ANT induced a significant increase in chemotactic capacity (*p* < 0.01), which is consistent with the RTCA results (Figure 2(e)).

### 3.3. The Copper Ion-Reducing Capacity of ANT Extracts

**Figure 3:**
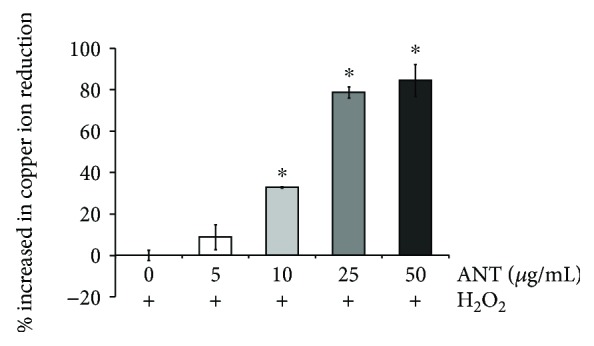
The Total Antioxidant Capacity (TAC) Assay for estimating copper ion reduction activity of the extracts under H_2_O_2_ stimulation. RDFs were treated with 5–50 *μ*g/mL ANT and stimulated with H_2_O_2_ for 24 h. The supernatant was used for TAC analysis. The mean values ± SD from three independent experiments are presented. ^∗^ *p* < 0.001 vs. H_2_O_2_ alone.

To confirm the antioxidant capacity of ANT against cellular damage, we examined their total antioxidant capacity and their copper ion reduction effect against H_2_O_2_ using the TAC Assay. We found that the copper ion-reducing capacity improved with the increase in ANT concentration in a dose-dependent manner (Figure 3). An extract concentration as low as 10 *μ*g/mL showed a significant increase in total antioxidant capacity compared with the control (32.96% ± 0.33, *p* < 0.001). Furthermore, 25 and 50 *μ*g/mL ANT showed an increase in the percent of copper ion reduction with the increase in ANT concentration (78.71% ± 2.79 and 84.56% ± 7.71, respectively, *p* < 0.001). Our results strongly indicated that ANT from *O. sativa* L. extract exhibit antioxidant capacity.

### 3.4. ANT Induce Collagen Type I Alpha 2 mRNA Expression and Suppress NF-*κ*B p50 and p65 mRNA Expression in H_2_O_2_-Stimulated RDFs

**Figure 4:**
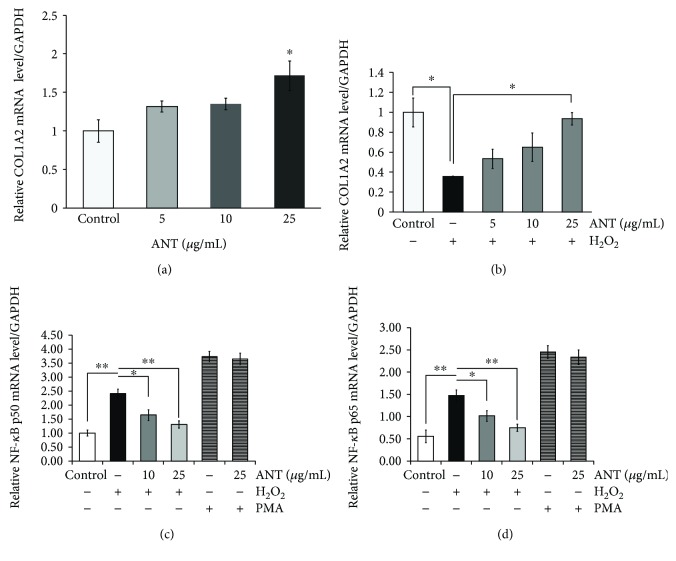
RT-qPCR analysis of the effects of ANT on H_2_O_2_-induced mRNA expression in RDFs at 10 h. (a) RDFs were treated with the indicated concentrations of ANT for 2 h, and then, the *COL1A2* mRNA levels were quantified. (b) RDFs were treated with the indicated concentrations of ANT for 2 h before the addition of 0.3 mM H_2_O_2_. *COL1A2* mRNA levels were quantified. Effects of ANT pretreatment on H_2_O_2_- or PMA-induced *NF-κB p50* (c) and (d) *p65* mRNA expression. The relative levels of mRNA were normalized against *GAPDH* from the same cDNA preparation. Values are presented as the mean ± SEM of three independent experiments. ^∗^ *p* < 0.05 and ^∗∗^ *p* < 0.01.

ANT affected the mRNA expression of *collagen type I alpha 2* (*COL1A2*), which has been determined to be upregulated when the skin is capable of regeneration. RDFs were incubated with 5, 10, or 25 *μ*g/mL ANT. RT-qPCR analysis showed that ANT significantly upregulated *COL1A2* mRNA at 25 *μ*g/mL (*p* < 0.05; Figure 4(a)). To further examine the effects of ANT on collagen synthesis under oxidative stress conditions, their action on cells stimulated with H_2_O_2_ was evaluated. The viability of H_2_O_2_-exposed RDFs was determined by the MTT assays. At 24 h, while 0.3 mM H_2_O_2_ did not alter the number of viable RDFs, an H_2_O_2_ concentration above 0.6 mM significantly decreased the population of viable RDFs (Supplementary Figure S1). Based on this assay, 0.3 mM H_2_O_2_ was used in further experiments. The presence of a low concentration of H_2_O_2_ suppressed the *COL1A2* mRNA level (Figure 4(b)). However, following 25 *μ*g/mL ANT pretreatment, the *COL1A2* mRNA level significantly increased (*p* < 0.05) compared with H_2_O_2_ stimulation alone (Figure 4(b)).

NF-*κ*B signaling plays an important role in inflammation and oxidative stress [31]. To investigate whether ANT reduce the upregulation of NF-*κ*B p50 and p65 subunits, RDF cultures were treated with H_2_O_2_ to induce transcriptional activation under oxidative stress. We assessed the mRNA expression of genes encoding these NF-*κ*B subunits using RT-qPCR. Compared with the control levels, H_2_O_2_ treatment for 10 h resulted in significant increases in the mRNA level of *NF-κB p50* (Figure 4(c)) and *p65* (Figure 4(d)), while treatment with H_2_O_2_ for 4 h had no effect on NF-*κ*B activation (data not shown). The addition of ANT prior to H_2_O_2_ treatment reduced the *NF-κB p50* and *p65* mRNA level induced by H_2_O_2_.

We also evaluated whether ANT influence the activation of NF-*κ*B directly or via ROS modification using PMA, which is an NF-*κ*B transcription inducer [32]. In this experiment, treatment with 40 ng/mL PMA induced almost 4-fold activation of NF-*κ*B expression (Figures 4(c) and 4(d)). In contrast to H_2_O_2_, a combination of ANT and PMA did not influence the inducing effect of PMA on both *NF-κB p50* and *p65* genes. In addition, ANT displayed a concentration-dependent inhibition of H_2_O_2_ stimulation of both NF-*κ*B subunits. These findings support the notion that cotreatment with ANT suppressed the H_2_O_2_-induced *NF-κB* mRNA level via the antioxidant effect rather than via direct inhibition of the NF-*κ*B pathway. Interestingly, the mRNA level of the NF-*κ*B subunits significantly and negatively correlated with the increased level of *COL1A2* in RDFs, suggesting a relationship between the collagen synthesis marker and the synthesis of transcription factors.

### 3.5. ANT Extract Protects Collagen Synthesis in RDFs from the Effects of H_2_O_2_

**Figure 5:**
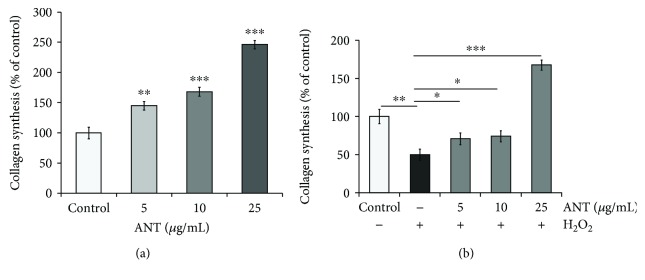
Effect of ANT on H_2_O_2_-stimulated RDF collagen synthesis. (a) RDFs were incubated with different concentrations of ANT for 24 h, and the effect on collagen synthesis was analyzed in terms of percentage change over the control. (b) Upon coculturing with various concentrations of ANT for 2 h with H_2_O_2_, collagen formation was measured in terms of percentage change compared with the control. Data are presented as the mean ± SD (*n* = 3). ^∗^ *p* < 0.05, ^∗∗^ *p* < 0.01, and ^∗∗∗^ *p* < 0.001.

Collagen biosynthesis-associated hydroxyproline was measured in RDFs that were treated with 5, 10, or 25 *μ*g/mL ANT and evaluated using a Total Collagen Assay Kit. ANT induced an increase in collagen biosynthesis in a dose-dependent manner (Figure 5(a)). At 25 *μ*g/mL, ANT caused approximately 2.5-fold induction of collagen formation compared with that in the control. Notably, the H_2_O_2_ group exhibited significantly decreased levels of collagen synthesis compared with the control (Figure 5(b), *p* < 0.01). The levels of collagen synthesis were significantly increased when RDFs were cocultured with H_2_O_2_ and 5 *μ*g/mL, 10 *μ*g/mL (*p* < 0.05), or 25 *μ*g/mL ANT (*p* < 0.001). In addition, pretreatment with ANT (25 *μ*g/mL) before H_2_O_2_ exposure induced ~3.4-fold induction of collagen formation compared with that in the H_2_O_2_-treated cells. These results suggest that ANT reverse the suppression of H_2_O_2_-induced collagen synthesis in RDFs.

### 3.6. ANT Effect on the Akt and ERK1/2 Signaling Pathways under Oxidative Stress

**Figure 6:**
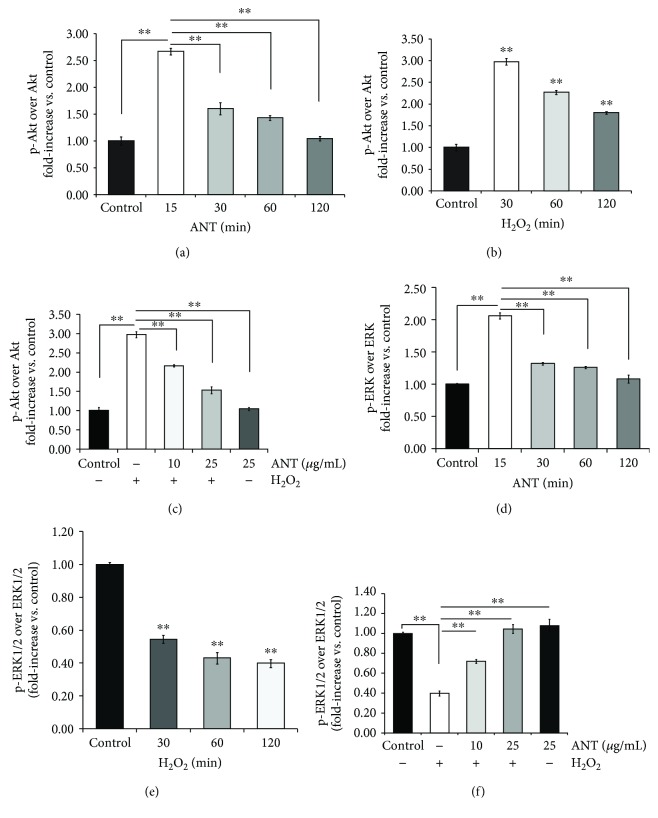
Involvement of the Akt and ERK1/2 signaling pathways in the antioxidative effect of ANT in RDFs determined by ELISA. Total and phosphorylated Akt expression after treatment with 25 *μ*g/mL ANT (a) or 0.3 mM H_2_O_2_ (b) for the indicated periods of time. RDFs were then pretreated with 10 or 25 *μ*g/mL ANT for 2 h and then incubated with 0.3 mM H_2_O_2_ for 30 min (c). Total and phosphorylated ERK1/2 expression after treatment with ANT (d) or H_2_O_2_ (e) under the same conditions as described for Akt. RDFs were then pretreated with 10 or 25 *μ*g/mL ANT for 2 h, followed by incubation with 0.3 mM H_2_O_2_ for 2 h (f). Results were analyzed by calculation of the phosphorylated protein relative to the total protein. Data are presented as the mean ± SD (*n* = 3). ^∗^ *p* < 0.01 and ^∗∗^ *p* < 0.001.

Protein kinase B (Akt) controls a wide variety of cellular processes including complex cellular programs such as differentiation, proliferation, and apoptosis and processes involved in immune responses [33]. Akt prompts NF-*κ*B activation through I*κ*B degradation [34, 35]. Herein, we revealed that ANT induced Akt phosphorylation as early as 15 min after the addition of ANT compared with the control (2.67 ± 0.06 vs. 1.00 ± 0.01, *p* < 0.001; Figure 6(a)).  Figure 6(b) shows that treatment of RDFs with 0.3 mM H_2_O_2_ induced ~3-fold Akt phosphorylation in 30 min (2.98 ± 0.08, *p* < 0.001). However, pretreatment with 10 or 25 *μ*g/mL ANT for 2 h was found to significantly decrease the level of H_2_O_2_-induced Akt phosphorylation in dermal fibroblasts at 30 min (2.17 ± 0.03 and 1.53 ± 0.09, respectively, *p* < 0.001; Figure 6(c)).

ERK1/2 signaling also has a prosurvival effect [36], and its activation is involved in the migration of skin fibroblasts [37]. Here, we showed the positive effect of ANT on fibroblast migration and then further explored the upstream regulator that protects dermal fibroblasts from oxidative stress. Thus, we investigated the effects of ANT on ERK1/2 phosphorylation under H_2_O_2_ exposure. ANT significantly induced ERK1/2 phosphorylation compared with the control (2.06 ± 0.05 vs. 1.00 ± 0.01, *p* < 0.001; Figure 6(d)). In contrast, 0.3 mM H_2_O_2_ incubation for up to 2 h suppressed ERK1/2 phosphorylation in a time-dependent manner, with the maximum effect observed at 2 h (0.40 ± 0.03, *p* < 0.001, Figure 6(e)). ANT pretreatment for 2 h at 10 or 25 *μ*g/mL effectively rescued the H_2_O_2_-induced ERK1/2 suppression (0.72 ± 0.02 and 1.05 ± 0.04, respectively, *p* < 0.001, Figure 6(f)).

### 3.7. Inhibitory Effects of ANT on the H_2_O_2_-Induced I*κ*B*α* Signaling

**Figure 7:**
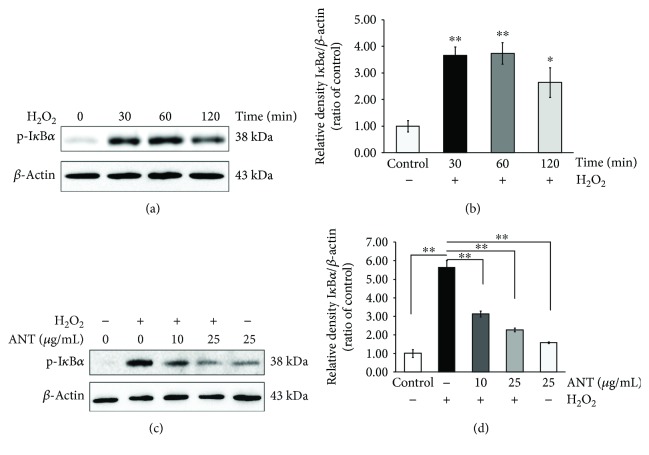
ANT inhibit the phosphorylation of I*κ*B*α* in H_2_O_2_-stimulated RDFs. Whole cell extracts were taken and assayed using western blotting. (a) RDFs were stimulated with 0.3 mM H_2_O_2_ for the indicated time points to evaluate the I*κ*B*α* phosphorylation status. (b) Data are expressed relative to untreated cells. (c) RDFs were pretreated with 10 or 25 *μ*g/mL ANT for 2 h, followed by the absence or presence of 0.3 mM H_2_O_2_ for 1 h. (d) Data are expressed relative to untreated cells. *β*-Actin was used as a loading control. Results are expressed as means ± SD of three independent experiments. ^∗^ *p* < 0.01 and ^∗∗^ *p* < 0.001.

As our previous experiment showed that the H_2_O_2_-induced oxidative response may depend on NF-*κ*B activity, we investigated the effect of ANT on this pathway. In the canonical NF-*κ*B pathway, the degradation of I*κ*B*α* protein occurs after a signal-induced phosphorylation by I*κ*B kinase (IKK) [38]. This enables the translocation of the NF-*κ*B p50 and p65 subunits into the nucleus, resulting in the production and secretion of inflammatory cytokines [39]. Western blot analysis revealed markedly increased phosphorylation of I*κ*B*α* at Tyr42 within 30 min after H_2_O_2_ stimulation (Figure 7(a)). Quantification of these results showed a significantly increased expression of phosphor-I*κ*B*α* compared with the untreated cells (3.66 ± 0.325 vs. 1.00 ± 0.21, *p* < 0.001, Figure 7(b)), which decreased at the 2 h time point.

To determine whether ANT inhibit H_2_O_2_-induced I*κ*B*α* phosphorylation, we pretreated cells with ANT for 2 h and then exposed them to H_2_O_2_ for 1 h (Figure 7(c)). ANT-treated RDFs at 10 or 25 *μ*g/mL showed significantly reduced phosphorylation of I*κ*B*α* in response to H_2_O_2_ compared with H_2_O_2_ stimulation alone (5.63 ± 0.38 vs. 2.27 ± 0.10; Figure 7(d)). These results indicated that ANT inhibited H_2_O_2_-induced phosphorylation and degradation of I*κ*B*α* and thus the subsequent nuclear translocation of NF-*κ*B.

### 3.8. The Effects of ANT on the H_2_O_2_-Induced NF-*κ*B Nuclear Expression and Translocation

**Figure 8:**
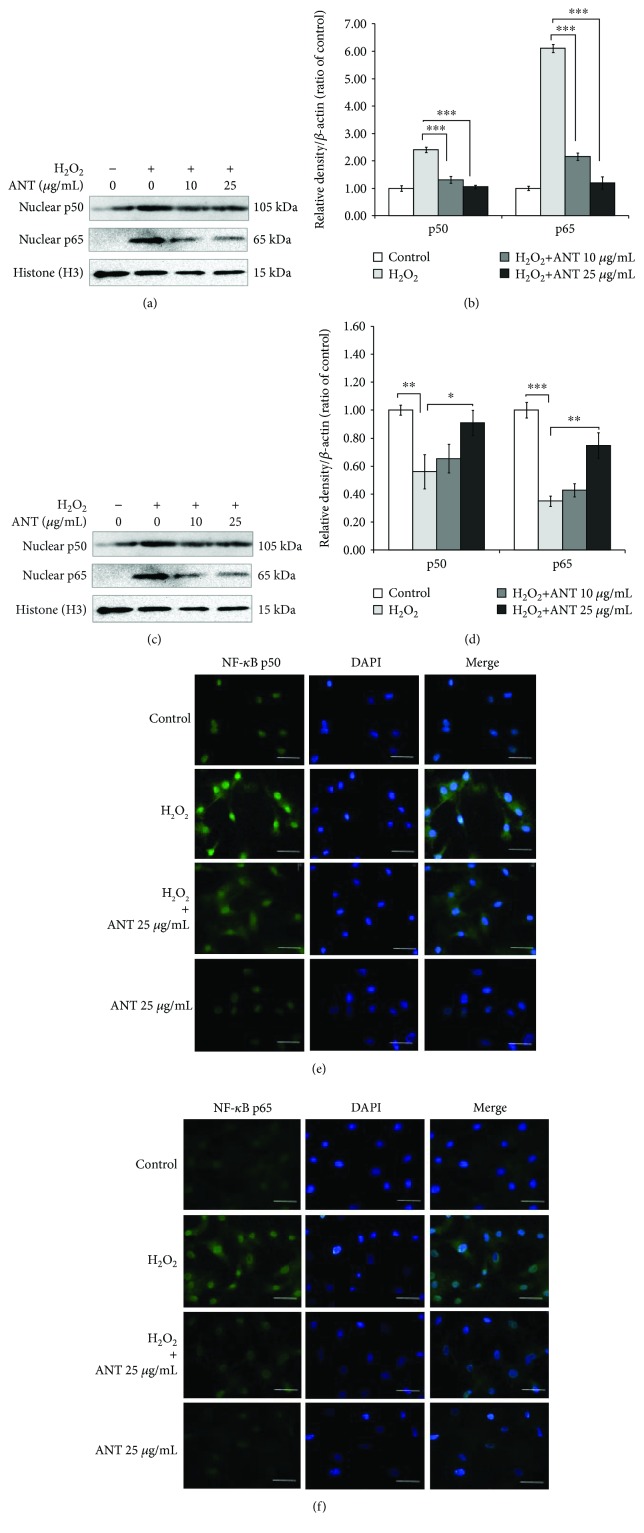
Effects of ANT on the activation of NF-*κ*B transcription factors in RDFs stimulated with H_2_O_2_. RDFs were treated with different doses of ANT for 2 h and then exposed to 0.3 mM H_2_O_2_. Ten hours after H_2_O_2_ exposure, cytoplasmic and nuclear extracts were subjected to western blot analysis. (a) Nuclear extracts were immunoblotted for NF-*κ*B p50 and NF-*κ*B p65. (b) Data are expressed relative to the control. (c) Cytoplasmic extracts from the same RDF preparations were immunoblotted for NF-*κ*B p50 and NF-*κ*B p65. (d) Data are expressed relative to the control. Histone H3 and *β*-actin were used as a loading control for the nuclear and cytosol fraction, respectively. Representative images of immunofluorescence staining showing NF-*κ*B p50 (e) and NF-*κ*B p65 (f) localization conjugated to FITC (green). Nuclei were stained with DAPI (blue). Scale bars: 50 *μ*m. Results are expressed as means ± SD of three independent experiments. ^∗^ *p* < 0.05, ^∗∗^ *p* < 0.01, and ^∗∗∗^ *p* < 0.001.

To confirm the effect of ANT on NF-*κ*B subunit-mediated transcriptional activation, further analysis of H_2_O_2_-induced NF-*κ*B expression in RDFs was performed. We examined the changes in NF-*κ*B p50 and p65 levels in the cytoplasmic and nuclear fractions. H_2_O_2_-induced RDFs showed an increase in NF-*κ*B protein in the nuclear fraction compared with the unstimulated control cells (Figure 8(a)). The NF-*κ*B expression upon H_2_O_2_ stimulation was significantly suppressed by 10 *μ*g/mL ANT (*p* < 0.001) and 25 *μ*g/mL ANT (*p* < 0.001) pretreatment in a dose-dependent manner (Figure 8(b)). Western blot analysis also showed that in normal conditions, the NF-*κ*B subunits localized in the cytosolic region of quiescent fibroblasts. However, after oxidative stimulation, the protein translocated into the nuclear region, resulting in a decrease in the expression in the cytosol (Figure 8(c)). ANT treatment resulted in significant inhibition of both NF-*κ*B p50 (*p* < 0.05) and p65 (*p* < 0.01) activation and translocation to the nucleus (Figures 8(a)–8(d)).

To confirm the cellular distribution of NF-*κ*B, immunofluorescence analysis was performed to investigate the effects of ANT on H_2_O_2_-induced NF-*κ*B translocation. Untreated RDFs showed a low signal of the cytoplasmic and nuclear staining of NF-*κ*B p50 Ab (Figure 8(e)). After H_2_O_2_ treatment, a marked nuclear staining was observed. However, ANT treatment prior to H_2_O_2_ stimulation decreased the level of nuclear translocation of NF-*κ*B p50 compared with that of cells treated with H_2_O_2_ alone. Similarly, there was an increase in the nuclear localization of NF-*κ*B p65 after cells were exposed to H_2_O_2_ compared with the control cells, and ANT treatment suppressed the localization of NF-*κ*B p65 in the nucleus (Figure 8(f)). There was no positive staining of the cells upon staining RDFs with the isotype-matched control IgG (Supplementary Figure S2). These findings further confirmed the results of the protein transcriptional activity in RDFs following free radical exposure and ANT pretreatment.

### 3.9. ANT Reduce IL-6 Production under Hydrogen Peroxide Stimulation in RDFs

**Figure 9:**
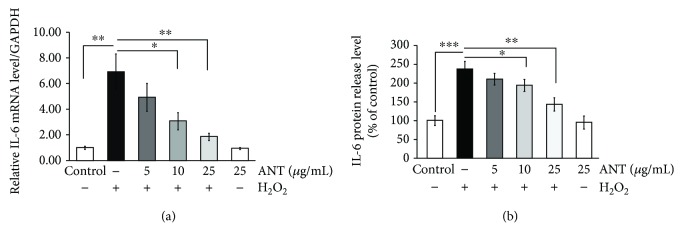
ANT reduce H_2_O_2_-induced interleukin-6 (IL-6) expression in RDFs. (a) RDFs were treated with different doses of ANT for 2 h and then exposed to 0.3 mM H_2_O_2_ for 10 h. *IL-6* mRNA levels were quantified by RT-qPCR. The relative levels of mRNA were normalized against *GAPDH* from the same cDNA preparation. (b) RDFs were exposed to 0.3 mM H_2_O_2_ for 24 h after a 2 h ANT pretreatment. The amount of IL-6 protein released into the cell culture supernatant following H_2_O_2_ treatment was measured by ELISA. The control values were arbitrarily set to 100%; all other samples are presented as percentage of control. Values are presented as the mean ± SD of three independent experiments. ^∗^ *p* < 0.05, ^∗∗^ *p* < 0.01, and ^∗∗∗^ *p* < 0.001.

Hydrogen peroxide activates the transcription process of various inflammatory mediators and cytokines [40]. To determine the consequences of NF-*κ*B activation, the mRNA transcription levels of *IL-6* were examined in H_2_O_2_-treated RDFs. H_2_O_2_ treatment induced the transcription of *IL-6* mRNA (6.92 ± 1.39 vs. 1.00 ± 0.12, *p* < 0.001, Figure 9(a)). However, when RDFs were pretreated with 10 or 25 *μ*g/mL ANT, the *IL-6* mRNA level was significantly decreased to 3.08 ± 0.67 (*p* < 0.05) and 1.86 ± 0.29 (*p* < 0.01), respectively. The protein released into the cell culture supernatant also increased after H_2_O_2_ treatment compared with the nontreated cells (236.37% ± 20.33 vs. 100% ± 13.01, *p* < 0.001, Figure 9(b)). Furthermore, a significant decrease in IL-6 protein release was found after pretreatment with ANT at 10 *μ*g/mL (193.36% ± 15.52, *p* < 0.05) and 25 *μ*g/mL (142.84% ± 17.63, *p* < 0.01) compared with the untreated control cells.

## 4. Discussion

ANT are members of the flavonoid group of phytochemicals, which have been shown to have substantial antioxidant effects, and are able to reduce lipid peroxidation and the deleterious effects of ROS [41]. Despite these findings, the preventive effects of black rice-derived ANT on wound healing and antiaging of skin have not been comprehensively examined. In this study, we initially performed a molecular target-based screening assay to identify the substance causing the induction of skin regeneration in rat primary dermal fibroblasts. We focused on the regulation of type I collagen expression by evaluating the level of *COL1A2* mRNA expression and determining the excreted proteins as well as the transcriptional regulation of genes involved in inflammation in RDFs. Herein, we report that ANT extracted from *O. sativa* L. induced collagen formation and prevented collagen degradation following H_2_O_2_ stimulation. The protective effects of ANT against cellular inflammation were investigated focusing on transcriptional activation of NF-*κ*B, which results from the inhibition of I*κ*B*α* phosphorylation [24]. Our study demonstrated that ANT inhibited the H_2_O_2_-induced nuclear translocation of NF-*κ*B p50/p65 heterodimers in primary dermal fibroblasts. Furthermore, ANT induced Akt/ERK1/2 phosphorylation and inhibited IL-6 expression in response to H_2_O_2_ stimulation. RDFs also exhibited cell migration capacities following ANT treatment.

The effect of exogenous oxidants highlights the importance of the balance among the proinflammatory signals that control skin repair. ROS generated by disturbance of the oxidation/reduction state of the cell have been implicated in the pathogenesis of various inflammatory diseases including skin damage. Our results demonstrated a protective effect of ANT extract by ameliorating the H_2_O_2_-induced cytotoxicity in primary dermal fibroblasts, which is consistent with previous studies indicating that the antioxidant activities of polyphenols can decrease oxidative stress by inhibiting ROS production and lipid peroxidation [42, 43]. Previous studies have shown that cell injury induced by H_2_O_2_ can be reversed by decreasing ROS generation; ANT extracted from red cabbage protected animals against oxidative stress by suppressing the NADPH-cytochrome-P450-reductase activity [44]. Cell injury causes inflammatory cells to migrate to the wound site and generate free radicals via a nonphagocytic NAD(P)H oxidase mechanism [15]. This causes an abundance of free radicals at the wound site. A measurement of NADPH-P450 reductase by its NADPH-cytochrome c reduction activity should be further determined in the black rice extract used in this study.

In our study, ANT were extracted in ethanol and contained molecules such as C3G, peonidin-3-O-glucoside, and quercetin-3-beta-D-glucoside. The total phenolic content was ~30 ± 1.5 mM gallic acid equivalent (gFW). Our results of the levels of C3G and the antioxidant properties of ANT extracted from *O. sativa* L. were lower than the levels in a previous study [26]. The differences in the C3G content and antioxidant capacity between the previous study and our findings probably stem from the diversity of rice cultivars, as well as the variety of extraction methods and analyses.

Numerous studies on the effects of natural or synthetic antioxidants on collagen deposition and on antioxidant defense have generated highly conflicting data, depending on the experimental system used. However, with various wound healing models, it has been repeatedly demonstrated that complex plant extracts containing active secondary metabolites (polyphenols, flavonoids, and alkaloids) [42, 43] and components of collagen-inducing polysaccharides, such as chitosan, and antioxidants such as curcumin [45] and resveratrol [46] ameliorated wound healing and increased skin collagen deposition, while suppressing proinflammatory markers. ANT extract from black soybean seed coats has been shown to decrease inflammation by suppressing the translocation of NF-*κ*B p65 into the nucleus of human dermal fibroblasts and keratinocytes [11]. Consistently, analysis of the effects of berries, including extracts and purified ANT, has revealed that ANT suppressed NF-*κ*B activation in monocytes and reduced the plasma concentration of proinflammatory mediators [47, 48]. In the wound healing mechanism, collagen type I plays a crucial role in maintaining the tensile strength and elasticity of the skin. Therefore, an approach that enhances type I collagen is an attractive strategy for dermal wound healing. ANT are the major active components in *O. sativa* L. extract, which may stimulate type I collagen biosynthesis. In this context, further clinical studies on the wound healing or antiaging activities of black rice extract should be performed *in vivo*.

Previous studies have reported that the main factor in UVA-induced damage to skin cells is the presence of ROS, including H_2_O_2_ and hydroxyl radicals, which affect the activation of the downstream cellular signaling pathways and cause damage to the skin [49]. The addition of H_2_O_2_ to a mammalian cell culture at concentrations of typically 50–1000 *μ*M induces growth arrest, cell death, and survival signaling pathways, including Akt [50, 51] and ERK1/2 signaling [52, 53]. Akt is a downstream target of NF-*κ*B and is involved in oxidative stress and inflammation [54]. ERK1/2 activation is associated with cell death induced by ROS [36]. However, signaling through ERK1/2 also has a prosurvival effect [55]. We demonstrated that H_2_O_2_ stimulated strong phosphorylation of Akt; however, ANT pretreatment suppressed this Akt signaling in a dose-dependent manner in RDFs. Under similar cell conditions, ANT prevented H_2_O_2_-induced suppression of ERK1/2 phosphorylation. The ERK/Akt pathways are involved in the differentiation of cells and the formation of collagen during tissue regeneration [56]. In addition, our study indicated that ANT induced collagen formation. Our results suggest that ANT can protect against ROS via modulations of the Akt/ERK1/2 signaling cascades, upstream of NF-*κ*B activation. Our investigations imply that ANT are a necessary requirement for collagen formation, which may occur via the ERK/Akt/NF-*κ*B signaling pathways. However, the molecular mechanisms that define the conditions for ANT-mediated ERK1/2 phosphorylation should be further investigated.

In aging skin, the NF-*κ*B signaling pathway may be attributable to the transcriptional control of collagen I gene expression in human skin fibroblasts [57]. NF-*κ*B is an early nuclear transcription factor that is triggered by many pathogenic stimuli such as oxidative stress. It is composed of various subunits, of which the p65/p50 heterodimer is the best known combination, and it has been shown to play a critical role in binding to a DNA consensus sequence to initiate the expression of proinflammatory genes [31, 58]. Several studies have demonstrated the role of NF-*κ*B in matrix metalloproteinases [59] and IL expression [60], which have been shown to be responsible for collagen degradation. In human dermal fibroblasts treated with H_2_O_2_, NF-*κ*B activity was induced; however, peptides derived from seaweed pipefish can suppress the NF-*κ*B activation thus decreasing the oxidative stress-mediated damage of the cells [61]. We observed that the H_2_O_2_-induced expression of p65 or p50 triggered an increase in NF-*κ*B activity accompanied by the downregulation of type I collagen expression in RDFs. Our results showing the declined transcriptional activity of *COL1A2* and type I collagen protein secretion in H_2_O_2_-stimulated cells suggest that NF-*κ*B affects *COL1A2* transcriptional activity. The inhibition of NF-*κ*B activation and induced collagen formation by ANT, which are among the best-described flavonoids, indicate an important role of ANT extract from the black rice in aging skin protection. Our investigation is in agreement with an *in vivo* study that demonstrated the anti-inflammatory role of ANT from Thai black rice using a 5-FU-induced oral mucositis model. This study indicated that ANT reduced the lesions induced by 5-FU in the oral mucosa via NF-*κ*B-mediated biological manipulations [26]. Furthermore, ANT suppressed the H_2_O_2_-induced IL-6 production and NF-*κ*B activation, which is in agreement with a previous study on normal human dermal fibroblasts exposed to UVB [62]. Our findings are consistent with previous observations in animal and clinical studies, where ANT demonstrated anti-inflammatory effects by inhibiting NF-*κ*B transactivation and suppressing proinflammatory mediators [48].

Several studies have reported that the dissociation of the p65-p50 NF-*κ*B heterodimer from phosphorylated I*κ*B*α* is accompanied by proteolytic degradation of I*κ*B. I*κ*B*α* degradation precedes NF-*κ*B activation, which in turn promotes I*κ*B*α* synthesis, restoring the unstimulated inhibited state [63]. To confirm the effect of ANT on oxidative stress-induced NF-*κ*B activation, the experiment with different time intervals of H_2_O_2_-induced NF-*κ*B activation and ANT pretreatment was conducted. Our results indicated that H_2_O_2_ induced tyrosine phosphorylation of I*κ*B*α*, which is in agreement with reports by Takada et al. [64] and Schoonbroodt et al. [65]. This favors our hypothesis that the intracellular levels of ROS control the level of phosphor-I*κ*B*α* by activating a kinase or inactivating a phosphatase that is specific to this protein. Therefore, the ANT-mediated low levels of ROS, by suppressing I*κ*B*α* phosphorylation, may abolish the specific proteolysis of phosphorylated I*κ*B*α* that induces NF-*κ*B activation. NF-*κ*B p50 phosphorylation at S337 is mediated by protein kinase A (PKA) [66] and promotes DNA damage-induced cytotoxicity [67]. In addition to PKA, NF-*κ*B p65 S276 phosphorylation is mediated by a number of other kinases and inhibits inflammation by regulating cytokine expression [68]. Our results showed that at 25 *μ*g/mL, ANT caused ~50% reduction in both NF-*κ*B p50 and p65 mRNA and protein expression in the nucleus, compared with cells exposed to H_2_O_2_ alone, suggesting that a combination therapy with other medicines may be required to fully affect NF-*κ*B transcriptional activity.

## 5. Conclusions

**Figure 10:**
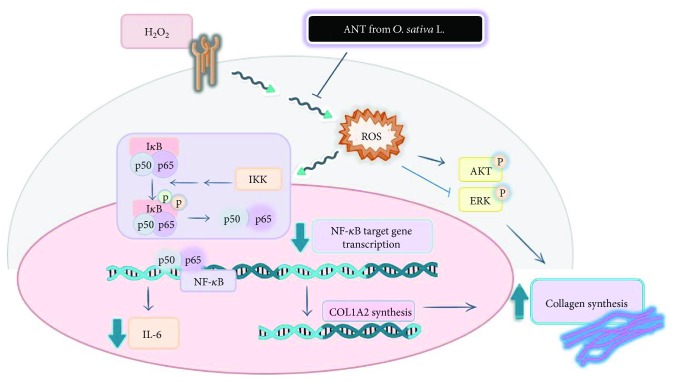
A schematic of the antioxidant and anti-inflammatory properties of anthocyanins (ANT) extracted from *Oryza sativa* L. in primary dermal fibroblasts. Black rice-derived ANT suppressed reactive oxygen species (ROS) signaling, which regulates phosphor-Akt/extracellular signal-regulated kinases (ERK) and NF-*κ*B p50/p65 signaling in inflammation, and modulated *collagen type I alpha 2* (*COL1A2*) upregulation. In addition, ANT suppressed hydrogen peroxide- (H_2_O_2_-) induced interleukin- (IL-) 6 production. The present study demonstrated that ANT may promote collagen synthesis in the skin and function as antioxidants and anti-inflammatory substances.

In conclusion, our data indicated that ANT extracted from black rice protected RDFs against H_2_O_2_-induced cytotoxicity by suppressing oxidative stress, confirming their antioxidative activity. ANT extract effectively upregulated the mRNA and protein expression levels of type I collagen in a dose-dependent manner. They also protected RDFs from H_2_O_2_-mediated inflammatory damage by inhibiting I*κ*B*α* phosphorylation, NF-*κ*B activation, and IL-6 production (Figure 10). We propose that ANT may be suitable as adjuncts in treatment promoting wound healing and antiaging. Further detailed validation studies are needed to investigate ANT as possible wound dressing materials, not only stimulating collagen synthesis but also accelerating wound healing by reducing oxidative stress.

## Figures and Tables

**Table 1:** Primer sequences used for RT-qPCR.

Target gene	Forward primer	Reverse primer	Amplicon size (bp)	Accession number
*COL1A2*	5′-ACCTCAGGGTGTTCAAGGTG-3′	5′-CGGATTCCAATAGGACCAGA-3′	222	NM_053356.1:1
*NF-κB p50*	5′-AGAGGATGTGGGGTTTCAGG-3′	5′-GCTGAGCATGAAGGTGGATG-3′	200	NM_001276711.1
*NF-κB p65*	5′-CGCCACCGGATTGAAGAAA-3′	5′-TTGATGGTGCTGAGGGATGT-3′	194	LC 369719.1
*IL-6*	5′-GCCCTTCAGGAACAGCTATGA-3′	5′-TGTCAACAACATCAGTCCCAAGA-3′	80	NM_012589.2
*GAPDH*	5′-CCCCCAATGTATCCGTTGTG-3′	5′-TAGCCCAGGATGCCCTTTAGT-3′	118	NM_017008.4

NF-*κ*B: nuclear factor kappa-B; COL1A2: collagen type I alpha 2; IL-6: interleukin-6; GAPDH: glyceraldehyde 3-phosphate dehydrogenase.

## Data Availability

The data used to support the findings of this study are available from the corresponding author upon request.

## Abbreviations

ANT: Anthocyanins
NF-*κ*B: Nuclear factor-kappa B
RDF: Rat dermal fibroblast
ERK: Extracellular signal-regulated kinase
Akt: Protein kinase B
MAPKs: Mitogen-activated protein kinases.
