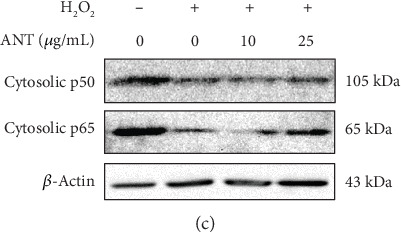# Erratum to “Antioxidant and Anti-Inflammatory Properties of Anthocyanins Extracted from Oryza sativa L. in Primary Dermal Fibroblasts”

**DOI:** 10.1155/2020/6306104

**Published:** 2020-07-05

**Authors:** Pakhawadee Palungwachira, Salunya Tancharoen, Chareerut Phruksaniyom, Sirinapha Klungsaeng, Ratchaporn Srichan, Kiyoshi Kikuchi, Thamthiwat Nararatwanchai

**Affiliations:** ^1^School of Anti-Aging and Regenerative Medicine, Mae Fah Luang University, Chiang Rai, Thailand; ^2^Department of Pharmacology, Faculty of Dentistry, Mahidol University, Bangkok, Thailand; ^3^Department of Pharmacology, Faculty of Medicine, Khon Kaen University, Khon Kaen, Thailand; ^4^Oral Tissues, Cells and Molecular Biology Analysis and Research Center, Faculty of Dentistry, Mahidol University, Bangkok, Thailand; ^5^Division of Brain Science, Department of Physiology, Kurume University School of Medicine, Fukuoka 830-0011, Japan

In the article titled “Antioxidant and Anti-Inflammatory Properties of Anthocyanins Extracted from Oryza sativa L. in Primary Dermal Fibroblasts” [1], the Western Blots in Figure 8(a) were duplicated in Figure 8(c) during the production process. The correct Figure 8(c) is shown below.

## Figures and Tables

**Figure 1:**