# Supplementary material for: Antioxidant and Anti-Inflammatory Properties of Anthocyanins Extracted from Oryza sativa L. in Primary Dermal Fibroblasts
Source: Oxid Med Cell Longev. 2019 Jul 31;2019:2089817. doi: 10.1155/2019/2089817 (PMC6701313; doi:10.1155/2019/2089817)
Supplement: Supplementary Materials — can be found at Supplementary Movie 1.gif and Supplementary Figures. [file 2089817.f1.zip › Supplementary materials.docx]

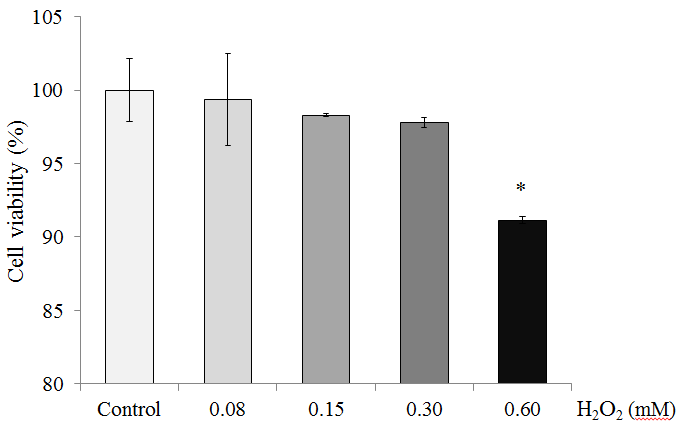


**Figure S1**. RDF cell viability challenged with H_2_O_2_ was measured using MTT assay. Cells were incubated for 24 h with H_2_O_2_. The mean values ± SD from three independent experiments are presented. **p* < 0.01 vs. control.


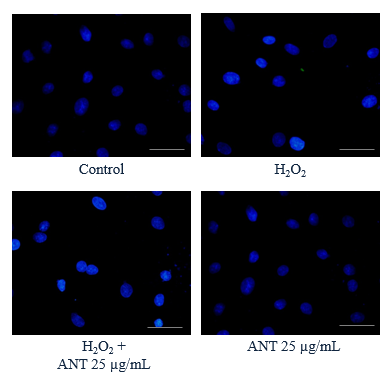


**Figure S2**. Representative images for immunofluorescence staining with isotype-matched control IgG on RDF after treatment with 25 μg/mL ANT with or without 0.3 mM H_2_O_2_. Nuclei were stained with DAPI (blue). NF-κB conjugated-FITC (green color). Scale bars: 50 μm.
